# COVID-19 Significantly Affects Maternal Health: A Rapid-Response Investigation from Pakistan

**DOI:** 10.3389/fgwh.2020.591809

**Published:** 2020-10-23

**Authors:** Inayat Ali, Salma Sadique, Shahbaz Ali

**Affiliations:** ^1^Department of Social and Cultural Anthropology, University of Vienna, Vienna, Austria; ^2^Department of Community Health Sciences, Peoples University of Medical and Health Sciences for Women, Nawabshah, Pakistan; ^3^Department of Anthropology, Pir Mehr Ali Shah (PMAS)-Arid Agriculture University Rawalpindi, Rawalpindi, Pakistan

**Keywords:** COVID-19, pandemic, reproductive health, mental health, low- and lower-middle-income countries, Pakistan, women, women's health

## Abstract

The coronavirus disease 2019 (COVID-19) is still unfolding. Its several implications are visible, yet more of them we have to observe and witness in future. Dealing with these impacts, this rapid-response article aims to situate the COVID-19 pandemic within Pakistan's overall sociocultural and politico-economic context; next to investigate the impacts of COVID-19 particularly the psychological ones on pregnant women in Pakistan via five case. One case history of Haleema (pseudonym) revealed how the pandemic exerted a substantial amount of mental pressure due to “arranging someone to accompany her to the hospital, finding a blood donor for her, and insecurity of convenience to hospital.” In this article, we show that Pakistan's geographical division into urban with an appropriate healthcare system, infrastructure and economic status, and more impoverished rural areas may show different impacts on people in general and the pregnant women in particular. This difference of facilities may contribute to disease transmission in the more deprived areas, that also due to cultural norms and mores such as shaking hands, cheek-kissing, and hugging that spread the virus are being overturned and that pregnant women are particularly vulnerable to psychological effects of the pandemic.

## Introduction

The coronavirus disease 2019 (COVID-19) is still unfolding. Although we have to observe and witness its several implications in future, its significant marks are already visible currently. It struck us when we learnt about Haleema—a pregnant woman in her last trimester living in a small village of Sindh province of Pakistan—who was struggling to find a female accompaniment to stay with her in the hospital ward for at least one night after the cesarean section and striving for a blood donor during the current 2020 COVID-19 global pandemic. This struggle is not exceptional during extraordinary times, and especially for those who live their lives below or around the poverty line.

Looking back at our sociocultural and biological history shows that infectious diseases have been challenging us for millennia. Historically, they have caused more morbidity and mortality than any other factor, including war (1). During the 1300s, the Black Death killed around one-third of the population of Europe within a few years (2). The Spanish Flu of 1918 killed between 20 and 100 million people (3). During the last two centuries, tuberculosis killed over a billion (4). In the 20th century, smallpox caused between 300 and 500 million deaths worldwide (5). In 2017, measles caused 110,000 deaths worldwide (6). Globally, from 1980 to 2018, vaccine-preventable diseases (VPDs) have affected around 1.66 million people (7). Until 2020, polio affects children in Afghanistan and Pakistan [Global Polio Eradication Initiative (8)].

Similarly, beginning in late 2019, the 2020 COVID-19 pandemic has spread far and wide. It gradually and rapidly transmitted from person-to-person, country-to-country, and continent-to-continent. At the time of this writing (22nd July 2020), infecting around 14 million people and causing over 610,000 deaths (9), the pandemic has significantly affected every facet of society at local, national, and global levels. The effects are multiple and differentially related to healthcare, physical well-being, mental health, sociocultural patterns, economy, and (geo)politics. Many healthcare systems are overwhelmed, even in high-income countries. Governments have introduced and implemented various measures to slow down the rapid escalation of the virus. Yesterday's “normal” attitudes and behaviors today seem “abnormal.” Despite our social nature, we are being recommended staying at home, observing isolation, and self-quarantine, and keeping a physical distance. These measures in some places are voluntary and in others are government-mandated and enforced. The greeting norms of the cordial handshake and, in some countries, hugging, bringing cheeks close, and symbolically kissing have become potentially deadly, resulting in a great deal of cultural confusion and the development of “air hug” and “leg-hugging” as new greeting rituals. Washing hands—sometimes up to 100 times a day—has also become part of “new normal.”

Concomitantly, COVID-19 has exerted substantial impacts on “at-risk” groups: older people, healthcare providers, children, the homeless, daily wage laborers, and the economically poor. Direct physical impacts of COVID-19 on pregnant women—that may result in pregnancy-related complications are still unexplored—in low-income countries, where various forms of inequalities and inequities considerably prevail. Yet the pandemic has indirectly had substantial effects on the health, specifically mental well-being, of pregnant women. Indeed, the virus can affect anyone, including the Prime Minister of the United Kingdom. Nonetheless, its effects are disproportionate. Who will contract the virus, and what kind of care they will receive are highly determined by socio-economic and political structures (Ali, 2020, under review)?

Anthropology has devoted substantial attention to the reproductive health (10–12); and the relationship between various forms of inequality and (re)emerging infectious diseases (13–18) to explore sociocultural, economic, and political factors that underpin health emergencies and positively shape the course of health interventions. It has been well documented that outbreaks of infectious diseases primarily affect the resource-deprived and disempowered people severely. The underlying reasons include “Malnutrition, dirty water, crowded living conditions, poor education, lack of sanitation and hygiene, and lack of decent healthcare provisions all increase chances that those who suffer from poverty will also suffer from infectious disease…. Crowded living and working conditions facilitate the spread of disease from person to person. Those who are poorly educated fail to take sufficient disease avoidance measures. Moreover, poor communities often lack adequate resources to improve sanitation” (19).

Yet, what implications an outbreak of infectious disease leaves on reproductive health is still not adequately explored terrain. Women in many countries, especially low-resource countries, suffer from socio-structural disparities and inequities due to a lack of economic resources and often cultural devaluation. These disparities considerably affect their reproductive health during “normal” times (20), then one can assume that the current challenging times can significantly affect pregnant women. Therefore, the aims of this rapid-response article are 2-fold: (1) to situate the COVID-19 pandemic within Pakistan's overall socio-cultural, political, and economic context; and (2) to investigate the impacts—specifically psychological implications—of COVID-19 on pregnant women in Pakistan via five case studies.

## Materials and Methods

The data for this rapid-response qualitative research come from several sources. First, for obtaining the first-hand and qualitative data on the impacts of the pandemic on pregnant women, we conducted five telephone interviews with pregnant women by using an interview guide. The interviews were conducted during March-April 2020 when the virus was steadily spreading in the country and the government was implementing several stringent measures, such as “lockdown.” Following the convenient sampling and sharing the aims and scope of this research among our family, friends, and acquaintances, we found five pregnant women. Afterward, we sent them the research protocols, consent form, and the interview guide. Once they agreed and gave their verbal ethical approval/consent, we called them via mobile phone to collect the required data. Second, we draw on our previous long-term ethnographic fieldworks in Pakistan, mainly in Sindh province—IA (2005–2011 and 2013–2020), (2013–2020), and (2012–2020)—to supply the qualitative data to offer the background information concerning the institutionalized forms of inequalities, and inequities, and perceptions and practices of health and illness. Each of us has conducted his/her research projects for masters and M.Phil. degrees, except Ali, who also has conducted his PhD research in the country, including the province. Third, we have done content and document analysis of the news reports and various surveys, mainly governmental reports, to contextualize the pandemic and situate its significant effects on pregnant women within this broader context. This paper is a part of the larger project approved by National Bioethics Committee of Pakistan (reference No.4-87/NBC-471-COVID-19-09/20/). Moreover, the names of interlocutors have been deliberately anonymized to maintain the necessary confidentiality.

## Background

### Pakistan: The National Context

Among the top ten most populated countries, Pakistan with an approximate total population of 212.82 million is at 150th position out of 189 countries on Human Development Index (HDI) (15, 21). One survey demonstrated that 10% of households did not have water, soap, or other appropriate cleaning agents in place for handwashing, around 70% had an appropriate sanitation facility,^1^ and 25% had flush toilets linked to a septic tank (22).^2^ Compared with 66% of men, merely 50% of women have formal education, and net (school) attendance ratio (NAR) is 59% at the primary and 38% at the middle/secondary level (15, 22).

Most of Pakistan's population perceives health and illness a divine intervention: health, illness, and recovery are predetermined (13–15, 23). As far as healthcare facilities and providers are concerned, in 2018, Pakistan had 1,280 public sector hospitals, 5,530 Basic Health Units (BHUs), 690 Rural Health Centers (RHCs), and 5,670 dispensaries (15, 21). Around a total of 220,850 registered doctors, 22,600 registered dentists, and 108,500 registered nurses are available in the county that give a current ratio of approximately: one doctor per 970 people, one dentist per 9,420 people, and one hospital bed for 1,610 people (15, 21). In terms of the provision of healthcare, rural populations have inadequate and inappropriate facilities than their counterparts (15). The Infant Mortality Rate (IMR), and the Maternal Mortality Rate (MMR) rates are considered higher than in neighboring countries and globally to be far too high: in 2015, IMR was 62/1,000, and MMR was 170/100,000 (21). Since 2003, around eight outbreaks of HIV have occurred in Pakistan that led the Joint United Nations Programme on HIV and AIDS (UNAIDS) to declare Pakistan the second fastest HIV- growing country across Asia (15). Other communicable diseases include malaria, polio, hepatitis, and measles still prevail here (13–15, 24). Being the two major contributors across the world, approximately the world's 80% of hepatitis affected people live in Pakistan and Egypt (15, 25). Due to neglected tropical diseases (NTDs), Pakistan is in the top 10 countries (26). And, in 2013, around 80 million people suffered from one or more chronic conditions such as cardiovascular diseases, cancers, diabetes, respiratory diseases, and mental disorders (27).

Pakistan has deep and prominent economic disparities, which further intensify based on gender and geographical area (15, 21). For gender-based inequalities and inequities, Pakistan was at 133 on the Gender Inequality Index (GII) in 2017 (15, 21). Recently, female participation in the labor market further decreased (21). In Pakistan, 67% of working women are engaged in the agriculture sector, 16% in the manufacturing sector, and 14.6% in community and personal services (21, 28). The overall unemployment rate of the country in 2017–18 was 5.79%, with high youth employment (21, 28). Around 24.3% of the country's population lives below the poverty line (earning US$2 per day) (15, 21). Moreover, 38% of children are “stunted” (short for their age), 7% were “wasted” (thin for their height), and 3% were overweight hefty for their height (15, 22). Women are also vulnerable to malnutrition, and micronutrient deficiencies resulting in pregnancy-related complications (29, 30). Around half of the women (52%) are overweight or obese (BMI ≥ 25.0), 5% of women age 15–49 are short (<145 cm), 9% are underweight (BMI > 18.5) (15, 22).

Moreover, cultural norms encourage communal patterns of living, especially in a joint or extended family: Three to four generations live together and share spaces that increase the frequency of physical contact (13, 15, 23). Average household members are around 7 (13, 22). Rural areas also include clusters of houses, locally called *Mohalla* or *Parro*, with one boundary wall, and one cluster may encompass around 100 members (13, 15, 23). Furthermore, cultural norms also encourage handshaking, hugging, and eating with hands—in part due to cultural mores but also to unaffordability of the required cutlery—and these norms regard not engaging in such behaviors as highly inappropriate and unethical (15). Likewise, many people, especially in rural areas, subsist on animals, and the economically marginal often share space with their cattle, including sleeping there at night (13, 15, 23). Not perceiving cattle, including their feces as unhygienic or harmful to health, these people drying dung to use it as a fuel for cooking food.^3^

Rumors and conspiracy theories are widespread in the country, specifically about vaccination programs, including a “Western plot” to sterilize Muslim women, vaccines have potential “side effects” that may kill children (13–15, 24, 34, 35). Although at the beginning of the pandemic suspicions about vaccination did not seemingly spill over into suspicion about COVID-19, currently conspiracy theories about COVID are circulating in Pakistan (14, 15, 24, 35). Some people believe that it is a “Jewish plot” to control the Muslim population, and doctors are working as agents of Jews (Salma et al., under review). One survey reveals that every fifth person in Pakistan believes that coronavirus is a conspiracy of international superpowers (36). In contrast, some people consider it as a “political game” to receive some financial aid (Salma et al., under review). Yet others are spreading rumors about how to deal with the pandemic, such as “brewing a black tea to drink five sips” (14, 24, 34, 35).

### Containing Measures: Government's Response During March–May 2020

Pakistan reported its first case on 26th February 2020, and now cases are rapidly increasing. When the cases in several countries are decreasing, there is a swift increase in the infections in Pakistan. By 18th May, the country reported around 41,000 confirmed cases and 900 deaths (9). Drawing an overall picture of COVID-19 in Pakistan is crucial. Briefly described, the government implemented the following measures to deal with this virus. After rise of COVID-19 in China, Pakistan suspended flights to China, then to Iran, Qatar and Italy (15, 37). With no test kits available, the country sent specimens to China and the United States of America (USA) owing to the unavailability of test kits in the country, and later imported 1,000 kits from China (15, 37). On March 13, 2020, as the virus infected 30 people, the government closed educational institutions, shut the border with Afghanistan and Iran, opened a quarantine camp at the Pakistan and Iran border, banned congregations of people, including religious gatherings at mosques, churches and temples (38, 39). Although during the mid of March 2020, the country's Prime Minister ruled out the option of lockdown based on the information that 97% of COVID-19 patients recover (40), Sindh province had already implemented a lockdown during March 2020 (37). Thereafter, a countrywide lockdown was enforced after all, and quarantine centers were opened, especially in Sindh province (37). The police and the army were deployed across the country, including Sindh province, to enforce the containing measures, e.g., self-quarantine, physical distancing, shutting markets. If someone breaches these measures, s/he could be booked under Section 188 of the Pakistan Penal Code for violations of the ban: the penalty included 6 months in prison or a fine or both. Besides, people constantly heard via the media that they should stay at home. To help, the government started to distribute food items among daily wage laborers (15, 41).

In contrast, people criticized this distribution not just because of its low quantity but also due to (receivers) being photographed while receiving the food (with selfies) and shared on social media. Many people believed that the actual number of affected people was higher than those reported due to a lack of testing. Currently, the country is substantially in a phase to lift the months-old preventive measures: e.g., easing lockdown, opening shops, resuming domestic and international flights, and starting a domestic transport system. After illustrating the contextual information, now we would like to move to the case histories of pregnant women for demonstrating the impacts of the 2020 COVID-19 pandemic on them.

## COVID-19 and Reproductive Health: Five Case Histories Reveal Distinct Impact

### Case History 1: Severe Impacts of the Pandemic

I am Haleema, around 25 years old, in my last trimester. I am a house worker living with my husband's joint family consisting of 12 members in a small village in Sindh province. I received merely primary education, and my husband has done 10th grade standards. He works as daily wage labor in nearby mountains to load trucks and earns around US$2 a day.

Nowadays, I am fine, merely feel some dizziness, but have no flu, fever, or cough. Although I am living with my joint family in a *Parro* [more than one house with one boundary wall],^4^ I am not meeting someone else or going outside of the house. I go for C-section to deliver a baby, so there is no choice to give birth at home. Last time, I gave birth at a charity hospital. However, we have a family *Dai Aman* (lit. mother midwife—it is used for the traditional birth attendant (TBA)/midwife) who regularly visited me thereafter.

The current disease [COVID-19] has severely affected me, because this is my last trimester, and I already had one miscarriage prior to giving birth to my first son with a C-section. My delivery is complicated. During the last month, when I visited *Mandam* (gynecologist), she gave me a date of mid-April [we conducted this telephonic interview at the end of March]. The month is close. I am already feeling anxiety, dizziness, and a burden on my head. It seems my delivery date is soon.

Nevertheless, neither I can visit a clinic nor invite a *Dai Aman* to visit me for a checkup. Everyone is directing me to stay inside the home due to fear of contracting this disease. They do not permit me to go for a checkup due to the mentioned fears. There is a *Curshew* (curfew). I do not know what will happen. However, if my situation gets intense, then we will go to the hospital where I went the last time to give birth to my first child. It is a charity hospital; therefore, they do not ask about money. It is a neat and clean hospital. My family takes these decisions. When I am pregnant, they, especially my husband and her sister, take diligent care of me, and accompany me to the clinic for a routine checkup.

Due to our quest, we were in constant virtual contact with this family. A few days after the interview, we heard that they went for a checkup because she was feeling constant dizziness. It was quite challenging for them to visit a doctor, but after managing it, his husband brought her on a motorbike to that charity hospital. However, that hospital was situated close to an epicenter of coronavirus in Sindh province: Sukkur district. The distance between their village and the hospital is around 100 kilometers. During this visit, doctors asked them to come on the next day for a cesarean. This dizziness is her labor pain. We called again, and we found a complicated situation regarding a woman to stay with her at hospital, and to find a blood donner. This family called a family meeting to decide on searching for a suitable person. The following are the details with Haleem's husband, mainly his sister:

The hospital is near the epicenter of the disease. In this charity hospital, there will be many people coming from different areas, including other women, to give birth. Now, who should accompany her? She will need accompaniment because the doctors will keep her for a few days. And males are not allowed to go inside, except the hospital staff. Due to the Purdah system, a young woman or girl cannot accompany her. The older women are in the at-risk group for contracting COVID-19. Every family member is worried. Her husband can accompany her, but he cannot stay inside.

Second, she needs blood. This will be her third cesarean. For the first time, her father donated her blood. During the next delivery, some of their family members found some donors who voluntarily donated blood. This time, her father is old and cannot donate blood. Her husband is weak due to continuous working in the mountains, and has some underlying conditions; hence, he cannot donate the blood. And volunteers are difficult to find due to the ongoing lockdown and fear of contracting the virus. We cannot ask someone and put his [usually male members donate the blood, which is why we are using a masculine pronoun] life at a risk. The time is running short. There is still no blood donor. At the hospital, the blood is available, but we cannot buy it due to our economic unaffordability.

The very next day, we called again to family. They had somehow managed to find a 30-year-old woman to accompany her at the hospital, where she gave birth to a son by cesarean. Since they found no blood donor, her husband donated her blood. The hospital kept her for two nights and then discharged her. Her husband stayed outside the hospital and slept on the floor during both the nights. After 3 days in the hospital, they returned to their home.

### Case History 2: Preferences and Fears About Hospital

I am Husna, a 28-years old house worker with intermediate education. My husband has also the same educational qualification, who works as a tailor. Our monthly income is around US$100. I have two children and live in a joint family comprising of 10 members. I delivered both babies without any cesarean and did not have any miscarriage history. I do not visit any biomedical hospital for a normal checkup.

Presently, I am in the second trimester, and I do not suffer from any complications, such as flue, cough, and fever. I am not in quarantine as I do not believe that COVID 19 is a disease. It is propaganda. Thus, I am not in favor of quarantine or isolation. I am living at home and going outside as usual like a normal life.

I believe that coronavirus is only rumored by government and media. My deliberation is that “*Jese soch wese sehāt*“ (lit. your thoughts significantly affect your health). Thus, if you perceive corona as a disease, it will psychologically affect you. In my opinion, the coronavirus is not a disease; therefore, it cannot affect my health either directly or indirectly. Besides, I want to say to everyone, do not be panic about coronavirus as it is the wrath of Allah. Pregnancy period is the most important part of life, so just enjoy and do not take any stress during pregnancy.

However, the current pandemic has affected our economic position, as my husband is a tailor, and his shop is close. Economically, coronavirus is directly exerting severe effects on our life. We face many difficulties due to the closure of our shop. We belong to an economically low-income family. Despite that, we do not receive any governmental help, such as Funds from *Ihsas* and Benazir Income Support Program (BISP) [both are government supported initiatives to support the economically poor].

My first baby was born at a hospital. Yet I prefer to give birth at home than a hospital for three main reasons. First, I believe that home is better because giving birth at a hospital is too expensive economically, and many people like us could not afford high dues to the hospital for the treatment. For giving birth, we will call a *Dai*—a traditional midwife, who is an old and experienced woman and has been conducting deliveries for a long time. We do not pay money to her, but only give her a new dress and sweets.

Second, during the ongoing pandemic, the government wants to increase the number of infected COVID-19 patients: I am afraid that if I give birth at a hospital, and doctors mention my name in the list of COVID-19 patients.

Third, my husband's mother says me to give birth at home because in the past women, including her, used to do that: That was a preferred mode to deliver a baby.

Although I am in the second trimester, I do not receive any antenatal care. Because my husband's mother does not allow me to go for antenatal checkups at a hospital due to the current pandemic situation. Also, she prefers me to stay away from the hospital. In case, I have abdominal pain, I call the *Dai*, who then suggests some home remedies and does abdominal massage.

I think it is important to make decisions related to pregnancy by yourself because you are the person who is going through pain. However, in my case, my husband's mother decides about everything, including my health. My husband also recommends me to follow the advice of his mother as she is old and experienced who has faced these all situations too.

### Case History 3: Worries to Deliver a Baby at a Hospital

My name is Rimsha. I am 27 years old, usually live in Rawalpindi, Punjab, with my husband's joint family, but currently, I am in Karachi with my parents to give birth to my fourth child. I am a home worker and college graduate married to a shopkeeper. Our monthly income would be around US$550. I have three children and previously gave premature birth to a daughter, who died instantly. I am in my last trimester.

Currently, I am healthy with no cough, flu, or fever. By choice, after listening to news about the dangers of coronavirus and lockdown outside, I am staying with my family, not going outside to meet my friends. Back when things were normal, I used to meet my friends every month.

Usually, I go for a routine checkup. I have the authority to make such decisions. With my husband or mother-in-law, I visit a gynecologist. However, this month's visit is delayed because of the corona. COVID-19 has affected me not very much physically, but mentally it is disturbing, and I feel depressed.

I do not give birth to a child at home. It is always a cesarean. Because giving birth to a child at home is not possible, I must go to the hospital. My situation influences my choices.

My family members do not want me to visit a hospital during these days of quarantine. I am worried—how will I deliver a baby during these times of lockdown?

### Case History 4: Deep Psychological Pressures of the Pandemic

I am Subal, a 29-years old woman from Rawalpindi, Punjab, in my second trimester. I am a homemaker, have a college degree (12th-grade education), and live in a joint family. My husband, a university graduate, has a government job. Our monthly income is around US$400. I usually visit a clinic for routine antenatal care (ANC). Prior to this pregnancy, I have already delivered a baby via cesarean section and had two miscarriages.

Presently, I am healthy, *Alhamdullillah* (all praises for God), and have no symptoms of coronavirus such as flu, fever, or cough. Despite that, I am observing social isolation and self-quarantine. I stay inside my house and go nowhere outside. I spend the entire time with my family and kids. Observing these measures is by choice as we have been informed very much about the causes and consequences of coronavirus. This pandemic has not affected my health directly, but indirectly. Now I can't go outside for a walk and there is an enormous mental pressure. Everyone is worried. The entire day, the media discusses these issues.

Moreover, because I am a cesarean, it is not possible to give birth at home, but at a hospital that is safe place to give birth. There is a gynecologist where my family brings me to give birth. Hopefully, when I give birth, things will be improved. This virus will be gone.

Presently, we are psychologically very much under pressure. I'm not going for a checkup during this critical situation. Everyone is concerned and does not allow me to visit a hospital. However, when the situation is stable, I will go for a routine checkup—these decisions we all make together: my mother-in-law, my husband and me.

### Case History 5: Concerns and Anxiety to Deliver a Baby at a Hospital

My name is Rabia. I'm 33 years old, in my last trimester, and live in a joint family in Rawalpindi city of Punjab province. My husband and I have obtained bachelor's degrees. My husband is a government employee, and our per month income is around US$500. I had three miscarriages. I usually go for a checkup. The decision depends on my mother-in-law, but she respects my opinion and brings me to the hospital.

These days, although I often feel dizziness because this is the last trimester, I have no cough, flu, or fever. I don't go outside of my house. I physical social distancing, but sometimes my child stays close to me. All family members live together. We have revisited our hygienic patterns. The husband has brought anti-septic soaps, so we are regularly washing our hands. We are drinking green tea.

Thus far, COVID-19 has not affected our physical health, but it has exerted effects on our mental health. I will prefer to go to the hospital to give birth because I am cesarean. However, the current pandemic is disturbing us. May God protect us during these testing times!

My parents, husband and his parents are extremely cautious and worried that may Allah keep everything sane and safe. Days of delivery are near, and everything is under lockdown. We are fearful about what will happen in terms of going to a hospital and delivering a baby. There are news stories about healthcare workers being affected by the virus: what if someone is infected, who does my operation? What if we are infected, including my baby? I have no choice to give birth at home, hence, we pray that when I deliver a baby, everything is normal.

## Discussion

A few studies have already focused on the implication of COVID-19 on birth practices across the world (12, 42, 43). Yet the literature is scanter in terms of its geographical focus. Hence, this is the first study that has explored the early impacts of the ongoing pandemic on pregnant women in Pakistan and investigated the socio-cultural factors that are likely to facilitate its spread. These four case histories can be seen in the national context, where various forms of inequalities have persisted in since the country's independence in 1947. The case histories reveal prominent forms of inequalities and inequities between wives and husbands related to their education and work, and show a difference between rural and urban areas in terms of availability of healthcare facilities and easy accessibility to these facilities. In rural areas, one might have to travel far to reach a clinic or hospital. This lack of accessibility was further affected by the lack of affordability.

Like other epidemics, COVID-19 is most adversely affecting the economically poor and marginalized. In Haleema's case, because she and her family fall in the low-income category, they struggled to find the “right” woman who was not vulnerable to contract COVID-19 to accompany Haleema and stay with her in the hospital. The most pressing issue was to find a blood donor that during “normal” conditions (in the absence of COVID-19) would have been relatively more easier since many volunteers donate blood.

Through these five case histories, although none of the women contracted the virus, we can see its direct and indirect impacts on their lives and can note that these effects, the psychological, physical and economic, were more profound among the rural and low-resource settings. The three case histories, who were situated in the urban areas, had enough economic resources, were discussing psychological impacts of COVID-19. In contrast, Haleema, located in the rural area and belonging to a low-income family, shared apparent implications of the pandemic. Similarly, Husna's situation is resembling Haleema in terms of geographical location, economic position, and the significant economic impacts of the pandemic.

As we mention it in the title that this is a rapid-response article, who offers not enough evidence but provides ground to conduct further research studies on this important domain. These five case histories beg various questions: how many serious complications, including maternity-related deaths, could be directly attributed to COVID-19? What are the differences in the impacts of COVID-19 on the health of economically poor and rich, rural and urban women? How do Pakistani women's general socio-cultural disempowerment and subservience impact their healthcare and maternity-related choices? How does the situation in Pakistan differ from that in other countries, and how is it the same? The answers to these questions might add to already existing knowledge on outbreaks, epidemics, pandemics, and various forms of inequalities and inequities and their disproportionate effects and challenges, such as for the pregnant women.

## Study Limitations

Specific limitations encumber this study analogous to other studies. Geographical, financial, and time constraints restricted choosing sample methodology and size, as only five mothers were interviewed. The sample size is a great limitation of the study; hence, the results cannot be generalized across the country. Despite this limitation, these five case studies explore and provide the useful preliminary indication of the severe impacts of COVID-19 on pregnant women and how various inequalities and inequities related to healthcare and economic conditions, including those between urban and rural areas, play a role in critical and less critical implications. To some extent, this limitation of sample size is counterbalanced by our thorough overview of relevant content analysis regarding politico-economic disparities and by our earlier long-term ethnographic research works focusing on an interplay between sociocultural, economic and political factors that shape distinguishable perceptions and practices of health and illness in Pakistan. Considering the challenges posed by COVID-19 to conduct “traditional” ethnographic research, this rapid study holds great importance to contribute to the existing knowledge regarding maternal health, particularly from a geographical standpoint and provokes further in-depth studies to be conducted in the country.

## A Way of Conclusion

The ongoing COVID-19 pandemic, directly or indirectly, has affected almost everyone across the world, with multiple implications. In this rapid-response article, we have presented the first-hand data to situate this pandemic within Pakistan's overall cultural and socio-economic context and illustrated the longstanding socio-cultural norms, beliefs, and patterns that work to facilitate the spread of the disease. We have also shown the changing patterns and norms, as well as the government efforts, designed to hinder its spread, and demonstrated that the economically poor and disempowered rural population is most vulnerable both to infection and to lack of available treatment. The same is the case with pregnant women. Although any possible pregnancy-related complications due to COVID-19 are yet to be known, we have demonstrated that the pandemic is already exerting adverse social, psychological, emotional and economic effects on such women.

Interview data revealed that all interlocutors found it difficult to access a healthcare facility. The fact that people are saying they “choose” to stay home seems to show that they are claiming agency, even though they are being forced to stay home due to the imposed lockdowns, as we have seen, particularly Haleema suffered in this regard. Four interlocutors reported that the primary effect was on their mental health, due to the stress of worrying about: their births; their enforced inability to attend routine prenatal appointments or to go outside for fresh air and exercise, and contracting the disease. However, one interlocutor stated that the pandemic has only and significantly affected their economic position.

## Data Availability Statement

The datasets presented in this article are not readily available because The interlocutors have reservations about it. Requests to access the datasets should be directed to inayat_qau@yahoo.com.

## Ethics Statement

The studies involving human participants were reviewed and approved by The National Bioethics Committee Pakistan. Written informed consent for participation was not required for this study in accordance with the national legislation and the institutional requirements.

## Author Contributions

IA: conception and design, analysis, and drafting manuscript. IA, SS, and SA: data collection, interpretation, and proofreading. All authors contributed to the article and approved the submitted version.

## Conflict of Interest

The authors declare that the research was conducted in the absence of any commercial or financial relationships that could be construed as a potential conflict of interest.

## Footnotes

^1^An “appropriate sanitation facility” includes “any non-shared toilet of the following types: flush/pour flush toilets to piped sewer systems, septic tanks, and pit latrines; ventilated improved pit (VIP) latrines; pit latrines with slabs” (22).

^2^The survey followed stratified two-stage sample design. First, 580 clusters were selected, and in the second stage, which involved systematic sampling, a fixed number of 28 households per cluster was chosen—the total sample size was ~16,240 households (22).

^3^Annually, zoonoses (diseases transmitted from animals to humans) caused morbidity in billions of people worldwide and mortality in millions, (Organization) and thus were a significant public health concern (31, 32). Also, COVID-19 was a zoonotic disease (33). Nevertheless, these animal-dependent people had no or little choice about their level of proximity with their animals.

^4^This is our translation.
